# Luteolin exerts an anticancer effect on NCI-H460 human non-small cell lung cancer cells through the induction of Sirt1-mediated apoptosis

**DOI:** 10.3892/mmr.2015.3956

**Published:** 2015-06-18

**Authors:** LIPING MA, HONGJUN PENG, KUNSHENG LI, RUNRUN ZHAO, LI LI, YILONG YU, XIAOMING WANG, ZHIFENG HAN

**Affiliations:** 1 Department of Thoracic Surgery, The Second Affiliated Hospital of Nanjing Medical University, Nanjing, Jiangsu 210003, P.R. China; 2 Department of Pediatrics, Jinling Hospital, Nanjing University School of Medicine, Nanjing, Jiangsu 210002, P.R. China; 3 Department of Thoracic Surgery, BenQ Medical Center, Nanjing Medical University, Nanjing, Jiangsu 210019, P.R. China

**Keywords:** luteolin, non-small cell lung cancer, Sirt1, apoptosis

## Abstract

Luteolin is a falconoid compound, which exhibits anticancer properties, however, its contribution to Sirt1-mediated apoptosis in human non-small cell lung cancer remains to be elucidated. The present study confirmed that the anticancer effect of luteolin on NCI-H460 cells was through Sirt1-mediated apoptosis. The NCI-H460 cells were treated with different concentrations of luteolin, and a 3-(4,5-dimeth yl-2-thiazolyl)-2,5-diphnyl-2H-tetrazolium bromide assay, cell cycle analysis and annexin-V/fluorescein isothiocyanate and propidium double staining were performed to assess the apoptotic effect of luteolin. Wound healing and Transwell assays were performed to confirm the inhibition of NCI-H460 cell migration. The protein levels of Sirt1 were knocked down in the NCI-H460 cells using a lentivirus to further investigate the role of this protein, and the expression levels of the apoptotic associated proteins, Bad, Bcl-2, Bax, caspase-3 and Sirt1, were measured using western blotting. The results of the present study demonstrated that luteolin exerted an anticancer effect against NCI-H460 cells through Sirt1-mediated apoptosis and the inhibition of cell migration.

## Introduction

Lung cancer is becoming the leading cause of cancer-associated mortality worldwide, particularly in China (1,2). For patients with lung cancer, resistance to therapy is a common phenomenon, which threatens the success of the treatment currently used against the disease. Therefore, novel theraperutic strategies are required for the overcome tumor evasion.

Flavonoids are known for their wide spectrum of pharmacological properties, including antioxidant, antimicrobial and anticancer effects (3). Luteolin (3′,4′,5,7-tetra-hydroxyflavone) is a common dietary flavanoid, which, similar to several other flavanoids, exists in several traditional Chinese medicines (4). Luteolin has been demonstrated to exhibit anticancer properties, including the induction of apoptosis and cell cycle arrest, and the inhibition of metastasis and angiogenesis, in several cancer cell lines, including the A549 non-small lung cancer cell (5).

Sirt1 is a well-known NAD^+^-dependent class III protein deacetylase, which belongs to the silent information regulator family (6). This family has multiple functions and is critically involved in the stress responses, cellular metabolism and aging, through the deacetylation of a variety of substrates, including p53, forkhead-box transcription factors, PGC-1α, NF-κB, Ku70 and histones (7,8). Sirt1 negatively regulates the tumor suppressor p53 and other tumor suppressors (9) and inhibits the transcription activity of AP-1 by targeting c-JUN (10). However, the possible roles of SIRT1 in the regulation of the NCI-H460 human lung carcinoma cell apoptosis have not been reported. The present study investigated the anticancer effect of luteolin on NCI-H460 by SIRT1 on the regulation of cell apoptosis. This finding provides novel insight into the mechanisms of luteolin's anti-lung cancer effects.

## Materials and methods

### Reagents

Luteolin was obtained from Sigma-Aldrich (St. Louis, MO, USA) and was dissolved in dimethyl sulfoxide (DMSO; Sigma-Aldrich) and adjusted to the final concentrations (20, 40, 80 and 160 µM) using complete RPMI-1640 medium. Paclitaxel (Taxol) was purchased from Haikou Pharmaceutical Factory Co., Ltd., (Haikou, China). The Taxol was diluted in serum-free culture media and was administered to cells at a final concentration of 300 nM. Fetal bovine serum (FBS), RPMI-1640 medium, Dulbecco's modified Eagle's medium (DMEM) and penicillin-streptomycin were purchased from Gibco Life Technologies (Grand Island, NY, USA). The 3-(4,5-dimethyl-2-thiazolyl)-2,5-diphnyl-2H-tet-razolium bromide (MTT) was obtained from Sigma-Aldrich. The primary and secondary antibodies used in the present study were obtained from Abcam (Cambridge, UK). All other reagents used were commercially available and of analytical grade.

### Cell lines and cell culture

The NCI-H460 human lung carcinoma cell line and HEK-293T cell line were obtained from American Type Culture Collection (Manassas, VA, USA) and cultured in RPMI-1640 and DMEM culture medium, supplemented with 10% heat-inactivated FBS, 100 U/ml penicillin and 100 µg/ml streptomycin at 37°C in a 5% CO_2_ incubator, respectively.

### MTT cell viability assay

Cell proliferation was determined using an MTT assay. Briefly, 1×10^4^ cells were seeded into 96 well plates and were treated with 0, 20, 40, 80 or 160 µM luteolin for 24 h at 37°C. Following treatment, the medium was replaced with fresh culture medium, containing 0.5 mg/ml MTT, and incubated for 4 h at 37°C. The culture supernatant was removed and the formazan crystals were dissolved in 150 µl DMSO for 10 min at room temperature. The absorbance was measured at 590 nm using an MK3 microplate reader (Thermo Fisher Scientific, Waltham, MA, USA).

### Wound healing assay

The NCI-H460 cells were seeded into a 6-well plate at 2×10^5^ cells/well and cultured until they reached 90% confluence. A single scratch wound was created on the confluent monolayers using a micropipette tip (1 mm), which touched the plate, as previously described (11). The wounded monolayers were washed with phosphate buffer saline (PBS) to remove cell debris, supplemented with serum-free medium, treated with 20, 40 or 80 µM of luteolin, and incubated for 24 h at 37°C. The cells migrated into the wound surface and the average distance of migrating cells was determined under an IX81 Olympus inverted microscope (Olympus, Tokyo, Japan) at 0 and 24 h.

### Transwell migration assay

Transwell migration assays were performed, as previously described (12). Briefly, the cells were seeded into a 24-well Transwell plates (Millipore, Bedford, MA, USA) in 10% FBS medium at a density of 1×10^5^ cells/well. Following incubation for 24 h at 37°C, the medium was replaced with serum-free medium and the cells were treated with 20, 40 or 80 µM of luteolin or 300 nM Taxol for 24 h at 37°C, while RPMI-1640 medium, containing 10% FBS was added to the lower chamber. The NCI-H460 cells were treated with the drug and cultured at 37°C for a further 5 h. The non-adherent cells were removed by washing with PBS, and the adherent cells were fixed in ethanol. Following staining with 0.1% crystal violet (Tokyo Chemical Industry, Tokyo, Japan), images were captured by microscopy (IX81; Olympus, Tokyo, Japan).

### Western blot analysis

The NCI-H460 cells were seeded into a 6-well plate at 3×10^5^ cells/well and were incubated with 20, 40 or 80 µM luteolin or 300 nM Taxol for 24 h, washed once with PBS and the cell lysates were prepared in radioimmunoprecipitation lysis buffer, containing 20 mM Tris (pH 7.5), 150 mM NaCl, 1% Triton X-100 and 1 mM PMSF. The protein concentrations were determined using a bicinchoninic acid protein assay kit (Beyotime Institute of Biotechnology, Shanghai, China). The proteins (30 µg) were resuspended in sample buffer containing 2% SDS, 2% β-mercaptoethanol, 50 mmol/l Tris-HCl (pH 6.8), 10% glycerol and 0.05% bromophenol blue. The proteins were separated on an SDS-polyacrylamide gel and transferred onto polyvinylidene fluoride membranes (Millipore). Following blocking of the membrane with Tris-buffered saline-0.1% Tween-20 (TBST), containing 2.5% bovine serum albumin, for 1 h at room temperature, the membrane was washed twice with TBST and incubated with primary antibodies overnight at 4°C. The primary antibodies were as follows: Rabbit anti-human anti-Sirt1 (1:1,000; cat. no. 2496), rabbit anti-human anti-Bad (1:1,000; cat. no. 4366), rabbit anti-human anti-Bcl-2 (1:1,000; cat. no. 2827), rabbit anti-human anti-Bax (1:1,000; cat. no. 5023), rabbit anti-human anti-caspase-3 (1:1,000; cat. no. 9665) and rabbit anti-human anti-cleaved caspase-3 (1:800). β-actin (1:1,000; cat. no. 8456) was used as an internal loading control. All antibodies were purchased from Cell Sigaling Technology, Inc. (Danvers, MA, USA). Following incubation with the primary antibodies, the membranes were washed three times for 10 mins each with TBST and were subsequently incubated for 1 h at room temperature with goat anti-rabbit immunoglobulin G horseradish peroxidase-conjugated secondary antibodies (1:5,000; cat. no. 7074; Cell Signaling Technologies, Inc.). Following washing three times for 10 mins each in TBST, the bands were detected using enhanced chemiluminescence reagent (Millipore). The band intensities were quantified using a ChemiDoc™ MP system (io-Rad Laboratories, Inc., Hercules, CA, USA).

### Reverse transcription-quantitative polymerase chain reaction (RT-qPCR)

The total RNA from the NCI-H460 cells was extracted using TRIzol reagent (Invitrogen Life Technologies, Grand Island, NY, USA), according to the manufacturer's instructions. The RNA concentration was determined using ultraviolet spectrophotometry (NanoDrop2000; Thermo Fisher Scientific, Waltham, MA, USA). cDNA was reverse transcribed from 1 µg total RNA using ReverTra Ace-α™ kit (Toyobo Co., Ltd., Osaka, Japan). A total of 2 µl template cDNA was used for amplification. RT-qPCR was performed using Thunderbird SYBR Master mix (Toyobo, Osaka, Japan). The primer sequences were as follows: Sirt1, forward 5′-AAGGAGCAAGGTCGCTTACAGA-3′ and reverse 5′-CAAATGGCTTTCAGATAGTCAGGTC-3′ and GAPDH, forward 5′-GATCCCGCTAACATCAAATG-3′ and reverse 5′-GAGGGAGTTGTCATATTTCTC-3′, and were all synthesized by GeneScript (Nanjing, China). RT-qPCR was performed on a Step One plus system (ABI, Carlsbad, CA, USA), using the following cycles: 95°C for 5 min and 40 cycles of 95°C for 15 sec, 60°C for 30 sec and 72°C for 30 sec. The expression of GAPDH was determined as an internal control. The 2^−ΔΔCT^ value was calculated for every sample and the mRNA expression levels were determined using the 2^−ΔΔCT^ method (13), normalized against GAPDH.

### Annexin V/propidum iodide (PI) flow cytometric analysis

Annexin V-fluorescein isothiocyanate (FITC)/PI double staining was performed to quantitatively determine the percentage of cells undergoing apoptosis. Briefly, the NCI-H460 cells were seeded into a 6-well plates at 2×10^5^ cells/well and at 70–80% confluence, the cells were treated with 20, 40 or 80 µM luteolin or 300 nM Taxol at 37°C. Following treatment for 48 h at 37°C, the cellular monolayer was released using trypsin without EDTA (Gibco Life Technologies, Carlsbad, CA, USA). The cells were resuspended and incubated with annexin V-FITC (KeyGen Biotech Co., Ltd., Nanjing, China) for 15 min at room temperature, followed by PI staining. The cells were analyzed using a flow cytometer (Becton Dickinson, Mountain View, CA, USA). Annexin V-FITC and PI double-negative cells were defined as normal cells, whereas annexin V-FITC-positive and PI-negative cells were defined as early apoptotic cells and annexin V-FITC and PI double-positive cells were defined as late apoptotic and necrotic cells. The annexin V-FITC-PI binding assay was determined at least three times. CellQuest software (BD Biosciences San Jose, CA, USA) was used to calculate the percentage distribution of normal, early apoptotic, late apoptotic and necrotic cells.

### Cell cycle analysis

To assess the cell cycle distribution, 2×10^4^ cells/well were seeded into 6-well plates, treated with 20, 40 or 80 µl luteolin and incubated for 24 h prior to analysis. The cells were fixed with 70% ethanol at 4°C overnight and were subsequently incubated with 100 µg/ml RNase A, and stained with 50 µg/ml PI at room temperature. The DNA content was determined using flow cytometry on a BD FACS Calibur flow cytometer (BD, Franklin Lakes, NJ, USA). The distribution of the cell cycles were analyzed using the ModFit LT software for Mac.

### Lentiviral vectors

A Sirt1 short hairpin (sh)RNA expression vector was constructed using pLentilHI, a lentiviral plasmid. The Sirt1 shRNA oligonucleotides (sense 5′-GATCCGGAT GAA AGTGAGATTGAATCAAGAGTTCAATCTCACTTT CATCCTTTTTTC-3′ and antisense 5′-TCGAGAAAAAAG GATGAAAGTGAGATTGAACTCTTGATTCAATCTCACT TTCATCCG-3′), corresponding to the 2,055–2,074 sites of the porcine Sirt1 mRNA (GenBank no. EU030283), were annealed and cloned into the pLentiHI at the *Bam*HI and *Xho*I sites (8).

### Lentiviral infection

The pLentiHI-Sirt1 shRNA transferred plasmid (3.3 µg), 2 µg Δ8.9 packaging plasmid and 3 µg VSV-G envelope protein plasmid were cotransfected into the HEK293T packaging cells (2×10^5^ cells/well) using Lipofectamine 2000 (Invitrogen Life Technologies), according to the manufacturer's instructions. Similarly, the FG30-Sirt1 or FG30 packaging plasmid and envelope protein plasmid (Takara, Shiga, Japan) were contransfected into the HEK293T packaging cells. Following transfection (48 h) at 37°C, the supernatant, containing viral particles, was collected and passed through a 0.45 µm filter (Merck Millipore, Darmstadt, Germany) to remove cellular debris. Porcine preadipocytes were seeded at a density of 1×10^5^ cells/well and cultured in DMEM/F12 medium, containing 10% FBS at 37°C. At 70–80% confluence, the viral suspension of Sirt1 shRNA and the scambled sequences, containing 6 mg/ml polybrene were added. Following infection for a further 48 h at 37°C, the cells were harvested for analysis.

### Statistical analysis

SPSS version 11.0 (SPSS, Inc., Chicago, IL, USA) for Windows was used for all statistical analyses. The data obtained from the different experiments are expressed as the mean ± standard error of the mean, from at least three independent experiments and were analyzed using Student-Newman-Keuls test. P<0.05 was considered to indicate a statistically significant difference.

## Results

### Antiproliferative effect of luteolin on NCI-H460 cells in vitro

**Figure 1:**
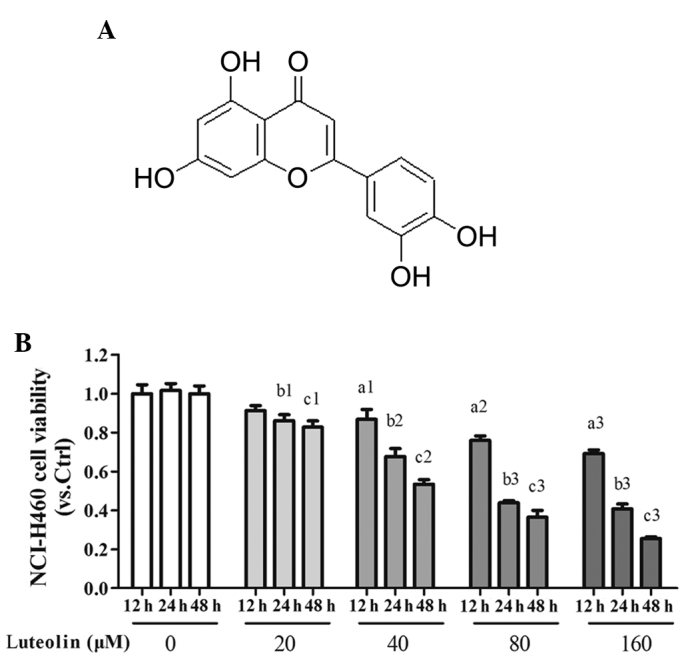
Effect of luteolin on the viability of NCI-H460 cells. (A) Chemical structure of luteolin. (B) Effects of luteolin on the viability of NCI-H460 cells were assessed using an MTT assay. The cells were seeded at a density of 1×10^4^ cells/well for 24 h. Following incubation, the medium was removed and the cells were washed three times with phosphate-buffered saline. Luteolin or serum-free culture medium was added into different wells and incubated for 24 h at 37°C. Following incubation, 20 µl 0.5 g/l MTT solution was added and incubated for an additional 4 h. Finally, the culture supernatant was removed and 150 µl dimethyl sulfoxide was added to dissolve the formazan crystals. Colorimetric determination was measured at 570 nm using a micro-plate reader. The data are expressed as the mean ± standard error of the mean (^a^compared with the Ctrl group at 12 h; ^b^compared with the Ctrl group at 24 h; ^c^compared with the Ctrl group at 48 h; ^1^P<0.05; ^2^P<0.01; ^3^P<0.001). Ctrl, control; MTT, 3-(4,5-dimethyl-2-thiazolyl)-2,5-diphnyl-2H-tetrazolium bromide.

The aim of the initial experiments was to investigate whether luteolin affected the viability of the NCI-H460 cell line *in vitro*. The range of concentrations assessed was between 20 and 160 µM. As shown in Fig. 1, treatment of the NCI-H460 cells for 24 h resulted in a concentration-dependent inhibition of the mitochondrial oxidative metabolism, determined using an MTT assay. The data revealed the inhibitor effect of luteolin on the viability of the NCI-H460 cells in a concentration-dependent manner. In the subsequent experiments, the NCI-H460 cells were treated for 24 h with three concentrations, 20 40 and 80 µM, which caused a reduction in cell viability by 86.06±9.23, 53.48±5.56 and 43.83±3.24%, respectively, in order to determine levels of apoptosis of the NCI-H460 cells.

### Effect of luteolin on the cell cycle and apoptosis of NCI-H460 cells

**Figure 2:**
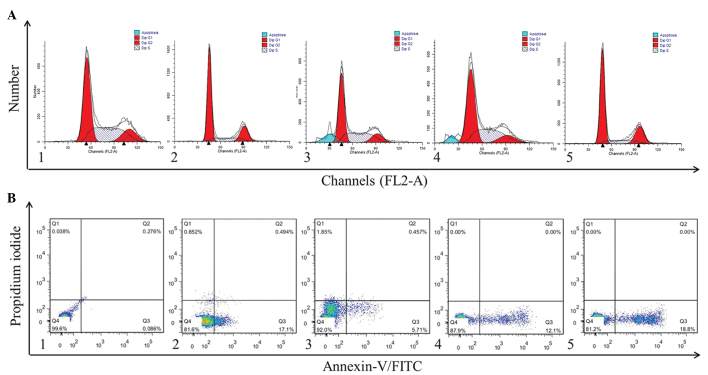
Cell cycle analysis and apoptosis detection in NCI-H460 cells. The cells were seeded into a 6-well plate and grown to 80% confluence in Dulbecco's modified Eagle's medium, supplemented with 10% fetal bovine serum. The cells were subsequently treated with different concentrations of drugs for 24 h. (A) Cells were harvested and fixed in 70% ethanol at 4°C overnight and were subsequently incubated with RNase A, stained with propidium iodide for cycle analysis, and the DNA content was determined using flow cytometry. (B) Cells were harvested and stained with annexin-V/FITC and propidium iodide, and the percentage of apoptotic cells was determined using flow cytometry. Luteolin redued the cell proliferation in NCI-H460 cells and this was associated with cell cycle arrest in S phase and induced apoptosis. ^1^Control group; ^2^300 nM Taxol group; ^3^20 µM luteolin group; ^4^40 µM luteolin group; ^5^80 µM luteolin group. FITC, fluorescein isothiocyanate.

The cell cycle distribution of the NCI-H460 cells was analyzed using flow cytometry. The effect of luteolin treatment for 24 h on cell cycle phase distribution is shown in Fig. 2A. A sub-G1 apoptotic peak was observed, which is usually regarded as one of the characteristics of apoptosis. Compared with the control cells, luteolin caused an accumulation of cells in the S phase (Fig. 2A). In addition, the apoptotic fraction was markedly increased following the addition of luteolin. These results demonstrated that the reduction observed in the proliferation of NCI-H460 cells mediated by luteolin was associated with cell cycle arrest in the S phase.

Subsequent investigation of the effect of luteolin on the apoptotic response of NCI-H460 cells, determined using annexin V-FITC/PI staining (Fig. 2B), revealed that the apoptotic ratio increased in the luteolin-treated NCI-H460 cells, compared with the cells in the control group. These results demonstrated that luteolin suppressed the proliferation of the NCI-H460 cells by inducing apoptosis.

### Effect of luteolin on apoptosis-associated proteins

**Figure 3:**
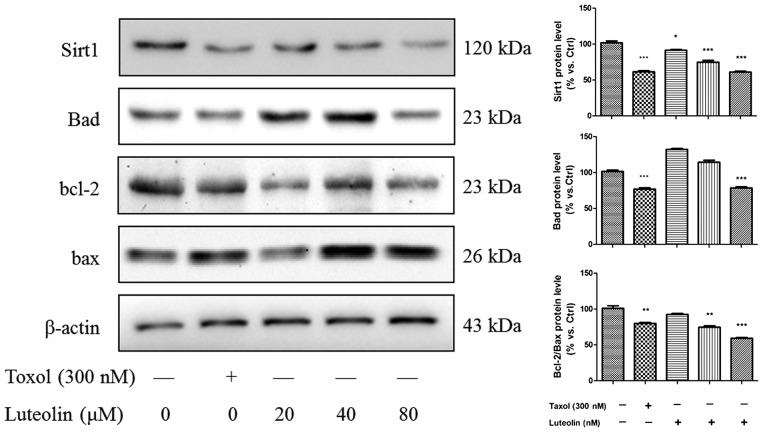
Assessment of apoptosis-associated proteins in luteolin-treated NCI-H460 cells. The cells were treated with luteolin for 24 h and the protein expression levels of Bax, Bcl-2, Bad and Sirt1 were assessed using western blot analysis. Data are expressed as the mean ± standard error of the mean^**^P<0.01 and ***P<0.001, vs. control group).

To confirm the effect of luteolin on apoptotic cell death, NCI-H460 lysate was extracted from the groups treated with different concentrations of the drug 24 h, and various apoptosis-associated proteins were analyzed using western blotting (Fig. 3). Luteolin increased the protein expression levels of apoptotic regulatory proteins, including the Bax/Bcl-2 ratio, in a concentration-dependent manner, however, only 80 µM luteolin inhibited the expression of Bad. Additionally, the expression of Sirt1 was analyzed in the presence of various concentration of luteolin. The results demonstrated that luteolin also decreased the expression of Sirt1 in the NCI-H460 cell line in a concentration-dependent manner.

### Effect of luteolin on NCI-H460 cell migration

**Figure 4:**
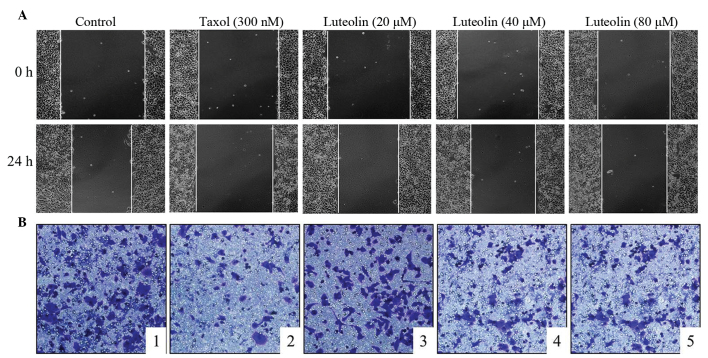
Effect of luteolin on the migration of NCI-H460 cells, determined using (A) wound healing and (B) Transwell migration assays. (A) Cells were seeded into a 6-well plate and grown to 90% confluence. A single scratch wound was generated on confluent monolayers using a micropipette tip. The cells were subsequently treated with the different drugs for 24 h and the migrated cells were observed under an IX81 Olympus microscope at 0 h and 24 h. (B) Cells were seeded into 24-well Transwell plates and treated with the different drugs for 24 h. Following treatment, the medium was replaced with serum-free medium and the cells were treated with different concentration of the drugs for 24 h. While the complete medium was added to the lower chamber, the cells were treated with the different drugs and cultured at 37°C for a further 5 h. Adherent cells were fixed in ethanol and stained using 0.1% crystal violet for visualization. The results demonstrated an inhibitory effect on the migration of the NCI-H460 cells (magnification, x200). ^1^Control group; ^2^300 nM Taxol group; ^3^20 µM luteolin group; ^4^40 µM luteolin group; ^5^80 µM luteolin group.

Cell migration is a hallmark of tumorgenesis and metastasis (14). It is relevant for angiogenesis to ensure tumor nutrition and for the formation of metastases, in which tumor cells leave the primary tumor site and spread to other tissues (15). Therefore, anticancer agents are required not only inhibit tumor cell growth, but also to prevent their metastases. As shown in Fig. 4, the NCI-H460 cell lines were treated with different concentrations of luteolin for 24 h and cell migration was assessed using a wound healing assay (Fig. 4A) and Transwell assay (Fig. 4B). The results demonstrated that luteolin exerted an inhibitory effect on the migration of the NCI-H460 cells.

### Sirt1 knockdown decreases the viability and induces the apoptosis of NCI-H460

**Figure 5:**
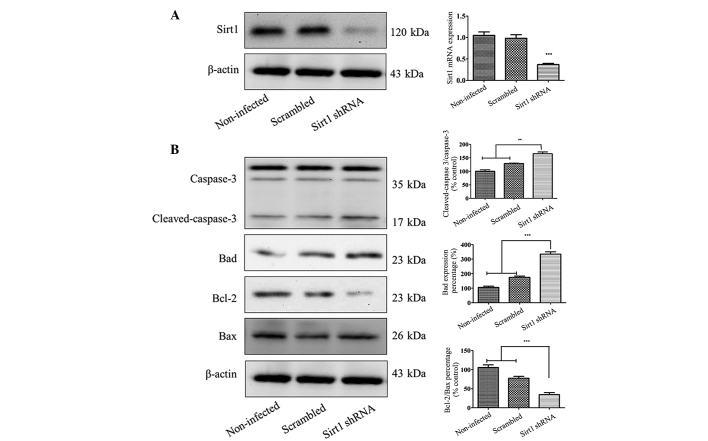
Sirt1 knockdown decreases the viability and induces apoptosis of NCI-H460 cells. (A) Sirt1 gene silencing was performed using shRNA in NCI-H460 cells. The NCI-H460 cells were infected with lentiviral supernatants containing Sirt1 shRNA or scrambled shRNA. Following infection (48 h), the expression of Sirt1 was determined using western blot analysis. (B) Effect of Sirt1 knockdown on the expression levels of caspase-3, Bad, Bcl-2/Bax was determined in porcine preadipocytes. Following infection (48 h), the expression levels of cleaved caspase-3 Bad, Bcl-2/Bax were detected using western blot analysis. The data are expressed as the mean ± standard error of the mean of three independent experiments (^**^P<0.01, ^***^P<0.001). shRNA, short hairpin RNA.

As the results suggested the potential role of Sirt1 in the regulation of NCI-H460 cell apoptosis, the present study hypothesized that the sensitivity to camptothecin may be increased by knockdown of Sirt1 using a lentivirus. The protein expression levels of caspase-3, Bad, Bax and Bcl-2 in the Sirt1-knockdown NCI-H460 cells were increased, compared with the empty vector and scrambled shRNA. As shown in Fig. 5A, Sirt1 shRNA significantly decreased the protein expression of Sirt1 in the NCI-H460 cells compared with the empty vector and scrambled shRNA. Following knockdown of Sirt1, the activation of caspase-3 in the NCI-H460 cells transfected with Sirt1 shRNA increased significantly, and the expression levels of Bad and Bcl-2/Bax were decreased, compared with the empty vector and scrambled shRNA groups (Fig. 5B). These findings suggested that Sirt1 regulated the apoptotic response of NCI-H460 cells.

## Discussion

The present study demonstrated that lueolin induced the apoptosis of NCI-H460 cells and downregulated the expression of Sirt1. It also demonstrated that the silencing of Sirt1 induced the apoptosis of NCI-H460 cells. Luteolin had an anticancer effect on NCI-H460 cells by affecting Sirt1-mediated apoptosis.

NCI-460, a human large cell lung cancer cell, is a subtype of non-small cell lung carcinoma (NSCLC), which is the most frequent type of lung cancer (16,17). Sirt1, a nicotinamide adenosine dinucleotide-dependent histone deacetylase, regulates the transcriptional activity of NF-κB (18). The role of Sirt1 and cortactin in unfavorable NSCLC characteristics and the progression of ADC has been described previously (10), and its function in the induction of cell apoptosis through the caspase-3 pathway in porcine preadipocytes has been demonstrated (8). However, whether Sirt1 is important in NSCLC remains to be elucidated.

Bax is a pro-apoptotic Bcl-2 family protein, which resides in the cytosol and translocates to the mitochondria upon the induction of apoptosis (19). Caspase-3 is a key terminal-regulated apoptosis protein involved in cellular apoptosis pathways. The present study demonstrated that Sirt1 knockdown increased the activation of caspase-3 and decreased the protein expression levels of Bad and Bcl-2/Bax, which induced the apoptosis of the NCI-H460 cells (6,20).

Luteolin is a flavonoid, which has been observed to exert anticancer activities, including the induction of apoptosis and cell cycle arrest, and the inhibition of metastasis and angiogenesis, in several cancer cell lines, including lung cancer cells (5,16,21,22). However, its effect on NCI-H460 cells remains to be fully elucidated. Therefore, the present study investigated the anticancer activity of luteolin on NCI-H460 cells. Luteolin demonstrated inhibition of NCI-H460 cell viability in a concentration-dependent manner, demonstrated using an MTT assay, and caused alterations in the cell cycle and induced apoptosis, determined using flow cytometry. Western blot analysis demonstrated that luteolin inhibited the protein expression level of Bad and the Bcl-2/Bax ratio, which can indicate the cell survival or apoptosis. In addition, luteolin inhibited NCI-H460 cell migration in a dose-dependent manner. These data indicated that luteolin may be a novel and effective anticancer agent and, at 80 µM, has an effect equal to that of 300 nM Taxol.

Notably, luteolin also inhibited the protein expression levels of Sirt1 in the NCI-H460 cell line. Therefore, it was suggested that the anticancer effect of luteolin on NCI-H460 cells was induced by Sirt1-mediated activation of the caspase-3 pathway. However, whether this effect is also induced by p53 and c-JUN requires further investigation (9,23,24).
